# Impact of Inequivalent Wetting on the Face-Specific Dissolution Rates for Single Faceted-Crystals Predicted from Solid-State Binding Energies

**DOI:** 10.1021/acs.cgd.2c00043

**Published:** 2024-06-10

**Authors:** Muhammad Najib, Robert B. Hammond, Tariq Mahmud, Toshiko Izumi

**Affiliations:** † Centre for Doctoral Training in Complex Particulate Products and Processes (CDT CP3), School of Chemical and Process Engineering, The University of Leeds, Leeds LS2 9JT, U.K.; ‡ Pfizer Research & Development U.K., Ramsgate Road, Sandwich, Kent CT13 9NJ, U.K.; § School of Chemical and Process Engineering, The University of Leeds, Leeds LS2 9JT, U.K.

## Abstract

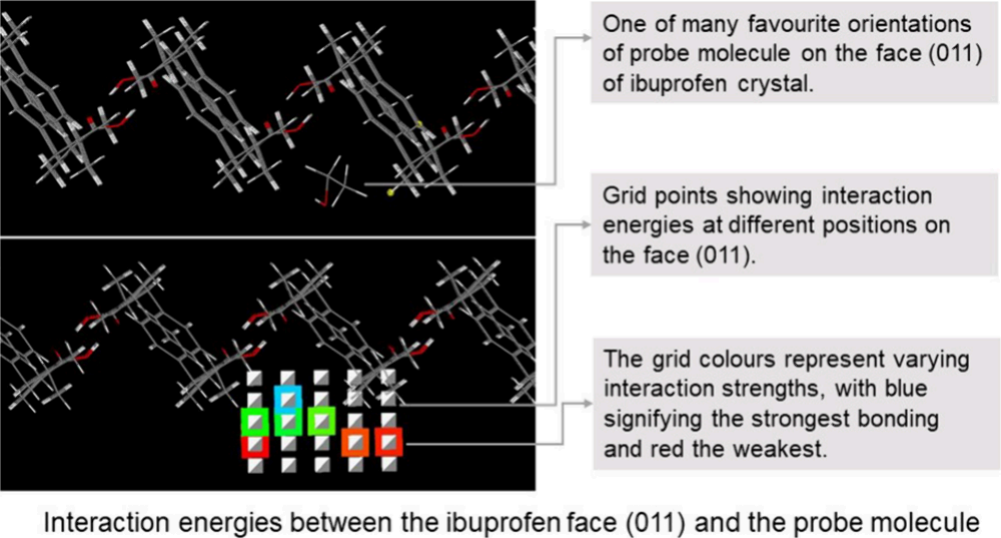

A methodology for the prediction of face-specific relative dissolution rates for single-faceted crystals accounting for inequivalent wetting by the solvent is presented. This method is an extended form of a recent binding energy model developed by the authors (Najib et al., *Cryst. Growth& Des*. 2021, 21(3), 1482–1495) for predicting the face-specific dissolution rates for single-faceted crystals from the solid-state intermolecular binding energies in a vacuum. The principal modification is that the equivalent wetting of the crystal surfaces is no longer assumed, since interactions between the crystal surfaces and the solution-state molecules are incorporated. These surface interactions have been investigated by using a grid-based systematic search method. The face-specific dissolution rates predicted by the extended binding energy model for ibuprofen in a 95% v/v ethanol–water solution and furosemide in an aqueous medium have been validated against the published experimental results and are in excellent agreement. This model is a step forward toward accurate predictions of the relative face-specific dissolution rates for a wide variety of faceted crystals in any dissolution medium.

## Abstract 2

A methodology is presented for predicting face-specific relative dissolution rates of single faceted crystals accounting for the inequivalent wetting by solvent. The predictions are validated against dissolution data for ibuprofen in ethanol−water and furosemide in aqueous medium with excellent agreement. It provides a step forward toward accurate predictions of dissolution rates for a variety of faceted crystals in different dissolution medium

## 1 Introduction

A detailed knowledge of the dissolution behavior of single crystals in solid dosage forms is crucial for their design, quality control, and therapeutic efficacy.^1^ There is a recent trend to understand the dissolution phenomena from the perspective of a single crystal solid-state molecular arrangement and interfacial interactions.^2−9^ The dissolution rate predictions based on the specific surface energies,^5^ and attachment energies,^10^ have failed to accurately predict the order of the dissolution rates from fastest to slowest dissolving faces. The relative propensity of a surface molecule to detach from a surface into a solution is an interplay of its interactions with the other molecules in the solid-state and in the solution-state.^4^ Therefore, both the crystal structure and interfacial interactions need to be considered for accurate dissolution rate predictions. The ability to predict the effect of solid-state and interfacial intermolecular interactions on the face-specific dissolution rates of a single crystal can be valuable to guide upstream crystallization to achieve an optimal crystal morphology.

In our previous article,^11^ a methodology was developed, referred to as the binding energy model, to predict the face-specific relative dissolution rates for single faceted-crystals, based on solid-state intermolecular binding energies in a vacuum. This binding energy model was successful in predicting the face-specific dissolution rates of ibuprofen in 95% v/v ethanol–water when compared with experimental measurements, but it gave a large discrepancy when predicting the dissolution rate ratio (101̅)/(001) for furosemide crystal-surfaces in water.^11^ This was mainly due to the inherent assumption of *equivalent wetting* in the binding energy model. It has been observed experimentally that changing the degree of undersaturation^10,12^ or the dissolution solvent^13^ changes the order of the fastest to slowest dissolving faces. Therefore, the effect of surface-solution interactions must be incorporated into the binding energy model to improve the accuracy of the predicted relative dissolution rates.

Molecular dynamics (MD) simulations have been used to predict the dissolution rates and dissolution mechanisms for different surfaces of crystals.^4,6^ However, MD simulations require significant computational time to produce physically realistic results and can be difficult to set up, requiring particular attention to attain properly equilibrated models. The results from MD simulations are usually more elaborate in terms of explaining the dissolution mechanisms compared to treating static models with molecular mechanics;^6^ however, the outputs, for example, the atomistic trajectories, require expert interpretation. Therefore, relatively simpler grid-based search methods, like the SystSearch algoritm,^14^ have been developed and applied successfully^15−19^ to study the effect of the solution-state molecules on the crystal surfaces.

This study aims to improve the binding energy model by carrying out the wetting analysis for ibuprofen and furosemide surfaces with their respective dissolution-medium molecules ethanol/water and water, respectively. A synthonic engineering tool, SystSearch,^20^ has been used to calculate the intermolecular interactions between different surfaces and solution-state molecules using the Dreiding II potential scheme.^21^ The SystSearch tool is a quick way of assessing the surface wettability by using only molecular structures of the host surface and the solvent probe molecule, and the results do not need any postprocessing.

The surface interactions between the ibuprofen faces (011) and (002) and the probe molecules, including ibuprofen, ethanol, and water, have been explored. The surface interactions between the furosemide faces (101̅), (010), and (001) and different probes including furosemide and water have also been calculated. The binding energy model was extended by incorporating surface interactions for both ibuprofen and furosemide. The relative dissolution rates predicted by the modified model are validated against the published experimental results for both model dissolution systems.^2,11^

## 2 Materials and Methods

### 2.1 Materials

Two material systems have been used, where the first is racemic (*R*)-(*S*)-ibuprofen (CCDC ref code: IBPRAC),^22^ in a saturated solution containing ibuprofen, ethanol, and water molecules. The experimental dissolution rates for the ibuprofen faces (011) and (002) in saturated 95% v/v ethanol–water solution were measured and presented in our earlier publication.^11^ The second system is furosemide form I (CCDC ref: code: FURSEM03)^23^ in an aqueous dissolution medium. The experimental dissolution rates for the furosemide faces (101̅), (010), and (001) in aqueous medium are available in the literature^2^ and have been used here for validation of the predicted dissolution rates.

### 2.2 SystSearch Method for Surface Interactions

The crystal surfaces are generated by atomistic modeling with a well-defined surface termination^19^ and no surface relaxation. A single reticular area (*S*_hkl_), representing the projection of a single unit cell is calculated from the slice thickness *d*_hkl_ and unit cell volume (*V*_cell_) as in eq 1.
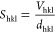
1

To minimize the effect of the reticular area edges, the slab thickness is generated with multiple unit cells as a 3 × 3 × 2 (*W* × *L* × *H*) matrix.^16,18^ Typically, a slab thickness of twice the interplanar spacing, *d*_hkl,_ is sufficient for capturing the interactions of the probe with the surface and subsurface molecules. There are instances when a slab thickness greater than twice the interplanar spacing should be used, for example, when the surface rugosity is large and the probe molecule can penetrate to a significant depth within the slab or for higher index faces when *d*_hkl_ is small. The direction perpendicular to the reticular area is aligned with the Cartesian *X*-axis, whereas the atomic positions in the *Y* and *Z* directions are represented by the respective fractional coordinates. A virtual grid of points is generated adjacent to the reticular area where step-sizes are defined to translate and rotate the probe molecule to capture the most favorable interfacial interactions. The translation normal to the surface is expressed in angstroms (Å), whereas the rigid-body orientation is controlled through a fixed angle step, usually 30° for rotations about the probe principal axes which are calculated from the atomic Cartesian coordinates. Therefore, the surface interactions with a probe are calculated at points on a six-dimensional spatial-rotational grid where the probe is rotated on each grid point and then translated to the next point until it visits every grid point to capture all the interactions stronger than the typical cut off energy value of −2 kcal/mol. The interaction energies are the summation of the interatomic interactions between the probe and the surface molecules according to eq 2.
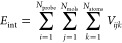
2where *N*_probe_ is the number of atoms in a probe molecule; *N*_mols_ is the number of molecules in a slab; and *N*_atoms_ is the number of atoms in the *j*th slab molecule.

**Figure 1:**
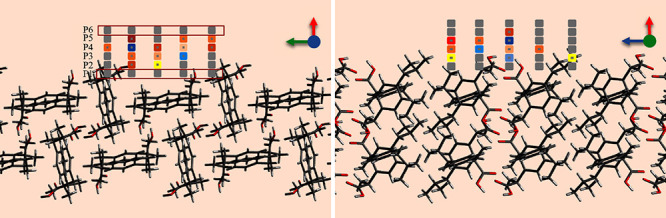
Grid points adjacent to a reticular area on the face (011) of ibuprofen, where (a) *uv* grid plane when viewing from the (+*w*) to (−*w*) direction; (b) *uw* grid plane when viewing from the (−*v*) to (+*v*) direction. The red, green, and blue arrows indicate *u*, *v*, and *w* directions, respectively. The molecular slabs were created in the Avogadro program.^24^ The grid points were generated for illustration in Microsoft Word.

The grid is adjusted in such a way that all the favorable interactions between the probe and the surface molecules are captured. Figure 1 is a schematic of the grid next to a reticular area on the face (011) of the ibuprofen with two viewing directions where image (a) is when viewed from the (+*w*) to (−w) direction and image (b) when viewed from the (−*v*) to (+*v*) direction. The slice visualization was generated from the .car file using the Avogadro program^24^ and grid points were created in Microsoft Word for illustration purposes. The colors have been used in the grid points to indicate that different strengths of interactions are captured on each grid point. The grid points have also been divided into layers as in Figure 1a, where P1 represents the first grid plane (inside the red rectangle) adjacent to the reticular area, whereas plane 2 is at a distance of 1 Å from the first plane. Similarly, there is a distance of 1 Å between each successive plane of grid points, and plane 6 is farthest from the reticular area. There are no colors on the grid points in the planes P1 and P2, indicating that no interactions were captured at these grid points, whereas the middle grid planes are colored, indicating that all favorable interactions have been captured.

### 2.3 Data Analysis Method

**Figure 2:**
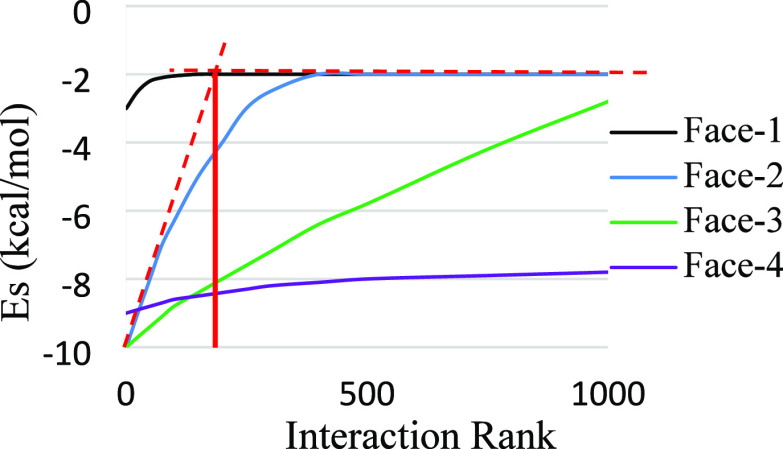
Schematic showing different interaction curves between a probe and five different faces. The curves vary due to the size and nature of the probe, the nature and orientation of the surface molecules, and the distance between the surface and the probe.

The surface interactions are arranged in descending order from strongest to weakest and then plotted as a function of the interaction rank^19^ (total number of intermolecular interactions). The interaction rank between different surfaces and probe molecules varies as a function of the nature of the solvent probe, the distance between the probe and the surface, and the characteristics of the surface; therefore, to reflect this, the trend lines also vary as shown in a schematic in Figure 2. The *x*-axis contains a rank of 1000 interactions, whereas the *y*-axis contains the surface interaction energies from −10 to −2 kcal/mol. The value of −2 kcal/mol represents the cutoff energy value, and any interactions less favorable than this are not expected to play a significant role in the dissolution process. The distribution curve for hypothetical face-1 represents very weak interactions, which quickly establish an asymptotic approach to a limiting interaction energy value, whereas the thousand most energetically favorable interactions for hypothetical face-4 are all strong and do not tend to the cutoff value of −2 kcal/mol. The interactions between large sized probe molecules and surfaces with high rugosity produce a large interaction rank, sometimes more than a hundred thousand. Since the distributions of interaction energies vary considerably between faces, a methodology has been adopted to estimate the interaction rank of the strongest interactions, which are expected to play a significant role in the dissolution process. The interactions falling within this interaction rank are averaged to represent the surface interaction energy value of that face with the respective probe molecule. The interaction rank which is used to average the strongest interactions varies for each face. A single interaction rank for all faces is not assigned, as it can produce misleading averaged surface interactions. For example, a singular interaction rank of 100 for the faces in Figure 2 will unnecessarily account for the weaker interactions for face-1, whereas it will not capture all of the strongest interactions for the face-4. Therefore, for each curve, a linear trend is fitted to the weak 5% asymptotic interactions, and then a best linear trend is fitted to the strongest interactions at the beginning of the distribution curve. The rank at the point of intersection of the two linear trends is used to calculate the average interaction energy. For example, linear trends are fitted to the interaction curve for face-2 in Figure 2, where the best linear fits to the asymptotic weakest interactions and the strongest interactions at the start of the curve are shown with red dotted lines, and a solid vertical red line from the point of intersection marks the interaction rank for averaging the strongest interactions.

### 2.4 Modified Binding Energy Model

The binding energy model from the previous publication^11^ of the authors based on the solid-state intermolecular interactions and the assumption of equivalent wetting of the crystal faces by the solution is reproduced in eq 3.
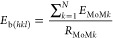
3where *E*_MoM*k*_ is the interaction energy between the reference molecule (Mo) at the center of the crystal and kth molecule on the face (*hkl*) of the crystal, which contributed greater than −0.01 kcal/mol to the lattice energy, and *R*_MoM*k*_ is the distance between the central reference molecule and the *k*th molecule on the face (*hkl*).

The relative dissolution rate (*R*_rel,D_) of the face *i* with respect to the face *j* was calculated according to eq 4.
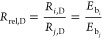
4where *R*_*i*,D_ and *R*_*j*,D_ are the dissolution rates of faces *i* and *j*, and *E*_b*i*_ and *E*_b*j*_ are the binding energies of faces *i* and *j*, respectively.

In order to modify the binding energies in eq 4 by incorporating the surface interactions between different faces of the crystal and the solution-state molecules, the most favorable (strongest) interactions between each face and each probe were averaged, according to the methodology described in Section 2.3. The solution-state molecules not only included the solvent molecules but also the dissolved solute molecules, which can also interact with the crystal surfaces during dissolution. The energy differentials (*E*_d_)^25^ were calculated to represent the relative strength of the solvent to solute interactions with different surfaces as given in eq 5.
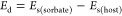
5where *E*_s(sorbate)_ is the the interaction energy between a surface (*hkl*) and a solvent probe, and *E*_s(host)_ is the the interaction energy between a surface (*hkl*) and a solute probe.

These differentials were used to predict the crystal growth morphology from a solution in a previous study.^25^ Several energy differential methods^18,25^ were tested in the present study, but the dissolution rates produced by the method in eq 5 were in better agreement with the experiments. The binding (*E*_b_)^11^ and the surface interactions (*E*_s_) energies were normalized by the respective molecular weights of the solute and solution-state probe molecules, respectively. Several approaches were used to normalize the surface interactions including the number of atoms of the probe molecules, molecular volume, and molecular weight. The dissolution rates predicted by the molecular weight normalization were in slightly better agreement with the experiments as compared with those of other approaches. The normalized *E*_s_ was used to modify the normalized *E*_b_ according to eq 6.
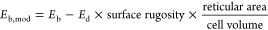
6where *E*_b, mod_ is the modified binding energy. Several methods^25,26^ to modify the attachment energy model in the literature are available to predict the crystal growth morphology from a solution, but not for the dissolution process. To modify the *E*_b_ to incorporate the effect of the solution, the geometric parameters related to the crystal structure and the surfaces, which were expected to play a significant role in the face-specific dissolution rate anisotropy, were identified such as unit cell volume, surface rugosity, and reticular area. Then similar to an existing modified attachment energy approach,^25^eq 6 was developed for the *E*_b,mod_ to predict the face-specific relative dissolution rates for single faceted-crystals. The face-specific relative dissolution rates were then predicted by eq 7.
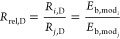
7where *E*_b, mod*i*_ and *E*_b, mod*j*_ are the modified binding energies of faces *i* and *j*, respectively.

## 3 Results and Discussion

### 3.1 Application of the Modified Binding Energy Model to Predict the Face-Specific Dissolution Rates of Ibuprofen

#### 3.1.1 Interaction Energies between Ibuprofen Surfaces and Probe Molecules

**Figure 3:**
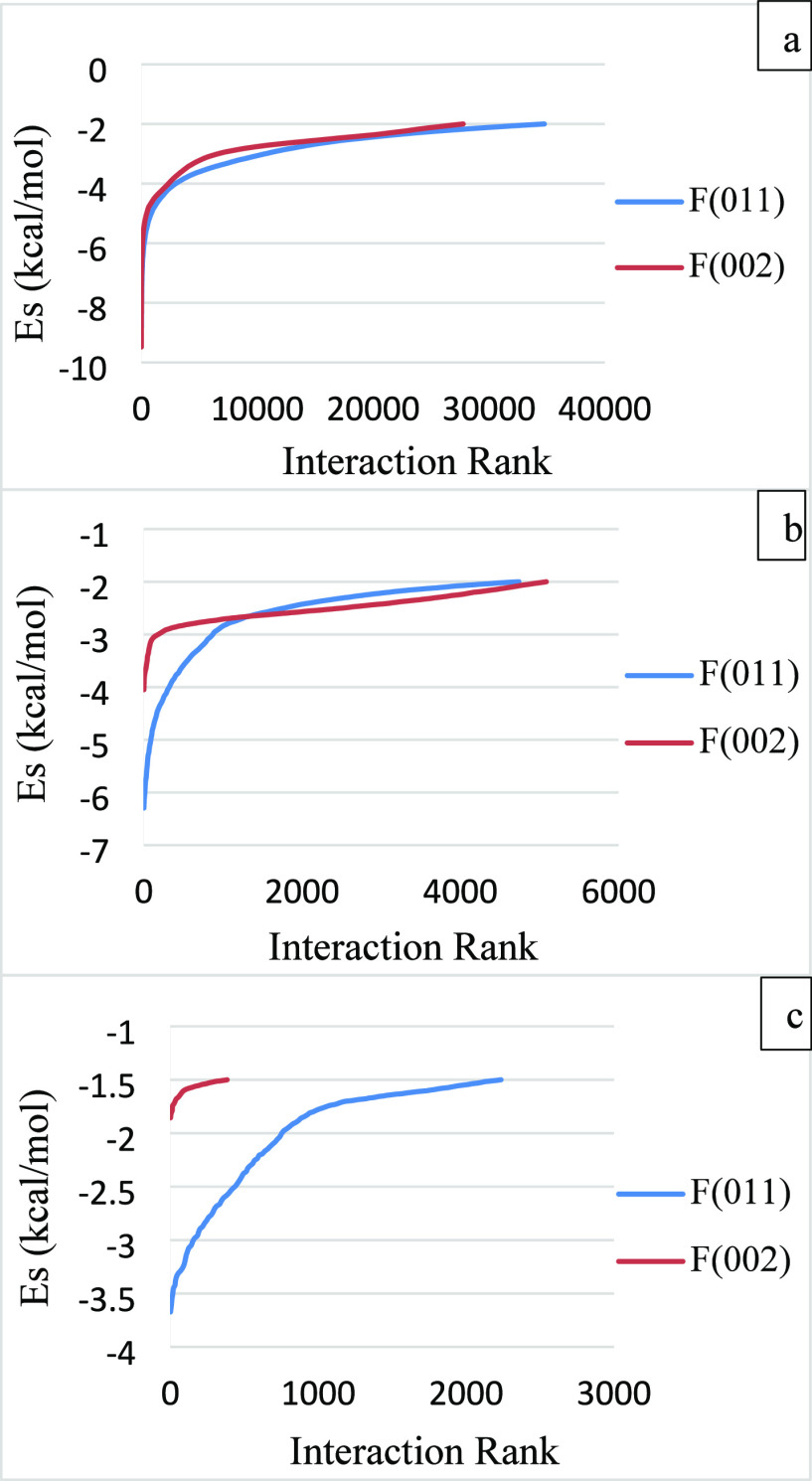
Interactions of the ibuprofen faces (011) and (002) arranged in descending order and plotted as a function of interaction rank for; (a) ibuprofen; (b) ethanol; and (c) water probes.

The interaction energies between the ibuprofen faces (011) and (002) and the probe molecules including ibuprofen, ethanol, and water are plotted as a function of the interaction rank in Figure 3. The trends in the interaction energy distributions between each probe molecule and the same crystal faces are different due to the different sizes and nature of the probe molecules. For example, the transition of the interactions from strongest to weakest is very smooth for the ibuprofen probe with face (002), whereas the transition is sharp for the same face with an ethanol probe. The interactions rank is a maximum for the ibuprofen probe, whereas it is smallest for the water probe. This huge difference is expected due to the difference in the size of the molecules and the carbon chain of the ibuprofen probe. Similarly, the interaction energy values are also different for each probe, where the ibuprofen probe has the strongest interactions with the faces (011) and (002), whereas the water probe has the weakest interactions with these surfaces. The interactions of the water probe with the faces (011) and (002) are relatively weak, so the cutoff value is reduced from −2 to −1.5 kcal/mol to capture some of the interactions for this study. The ibuprofen probe interacts with faces (011) and (002) quite similarly, and the change in the distribution curve from the stronger to weaker interactions is smooth, as illustrated in Figure 3a. This is due to the relatively larger size of the ibuprofen molecule and the longer carbon chain, which enables it to interact with the faces on most of the grid points. The trend of interactions between the ethanol probe and the faces (011) and (002) are different, where the transition from stronger to weaker interactions is smooth for the face (011), whereas for the face (002), the upturn from the stronger to weaker interactions is sharp. The water probe has a significant difference between its overall interactions with faces (011) and (002). The interactions with the face (002) are very few and very weak, whereas its interactions with the face (011) are relatively greater in number, but still not very strong compared to the interactions of face (011) with the ibuprofen and ethanol probes.

Figure 3 shows that the ibuprofen faces are more likely to bond strongly with ibuprofen and ethanol molecules as compared to the water molecules. The interactions of the faces (011) and (002) with the ibuprofen probe are calculated to accommodate the effect of the solution-state solute molecules. It is well-known that ibuprofen has low solubility in aqueous solutions^27^ and therefore belongs to BCS class II. The weaker interactions of the ibuprofen surfaces with a water probe indicate that strength of wetting is relatively small compared to ethanol solvent, which could be one of the various reasons for its low aqueous solubility.

#### 3.1.2 Favorable Orientations of Probe Molecules on Ibuprofen Crystal Surfaces

**Figure 4:**
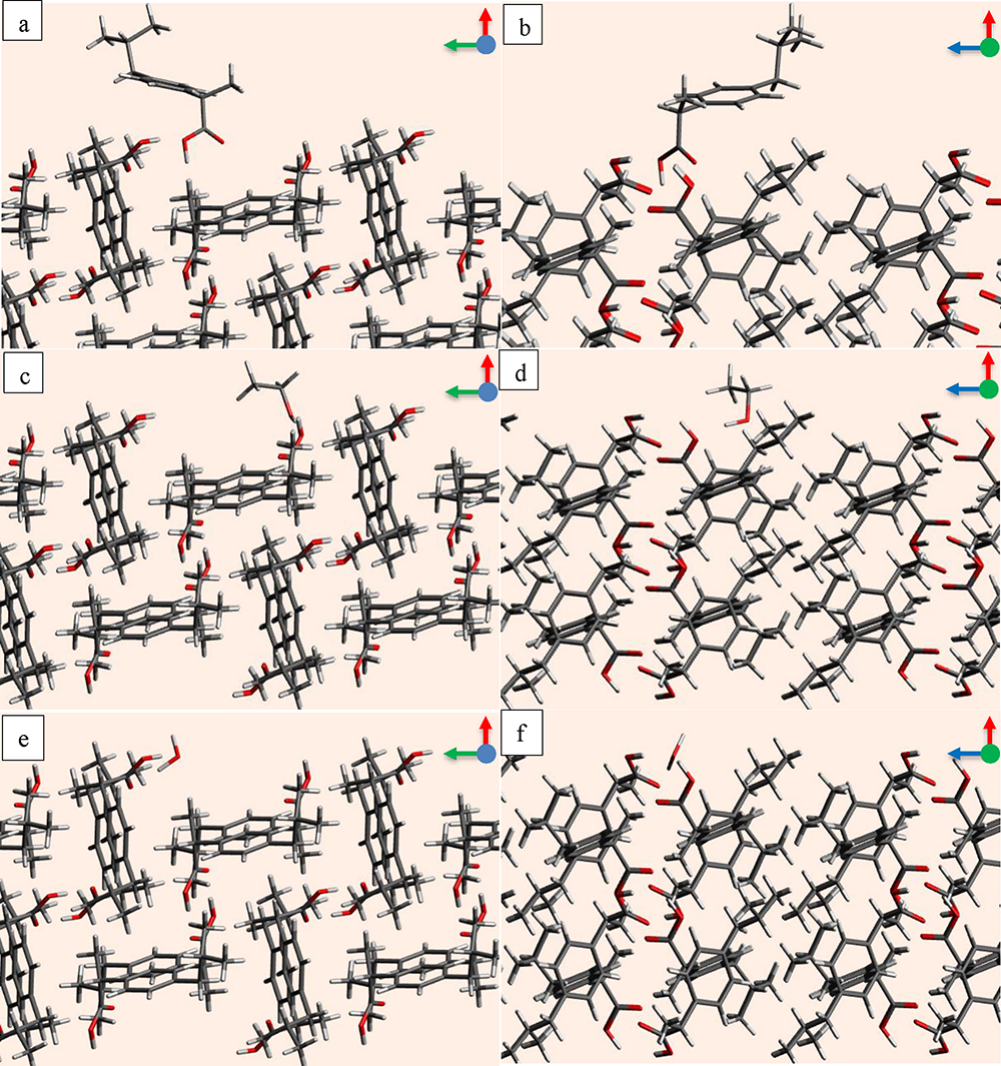
One of the many favorite probe orientations on the face (011), where column 1 (a–e) shows the *uv* plane when viewed from the (+*w*) to (−w) direction and column 2 (b–f) shows the *uw* plane when viewed from the (−v) to (+v) direction for (a, b) ibuprofen probe; (c, d) ethanol probe; and (e, f) water probe.

The probe orientation and position next to the face (011) to represent the favorite interaction was visualized using the Avogadro program, and examples are shown in two viewing directions in Figure 4. The ibuprofen probe projects its carboxyl group toward the carboxyl group of the ibuprofen molecules located on the face (011) to make strong hydrogen bonding interactions, whereas it tilts the tail end in a lateral direction to capture dispersive interactions as depicted in Figure 4a,b. Due to a large valley, as shown in Figure 4a, the ibuprofen probe has more options to get closer to the surface, and the two exposed carboxyl functional groups on the surface ensure stronger interactions. Many other orientations also produce stronger interactions, but only one is shown for each probe in Figure 4.

The ethanol probe projects its hydroxyl group toward the carboxyl group of the surface molecules to make strong hydrogen bonds, whereas its small tail also orients in a favorable direction to capture some dispersive interactions as shown in Figure 4c,d. The water probe also projects its hydroxyl group similar to ethanol and ibuprofen probes to capture strong hydrogen bonds, whereas due to lack of carbon chain unlike other probes, it is not possible to have strong dispersive interactions.

**Figure 5:**
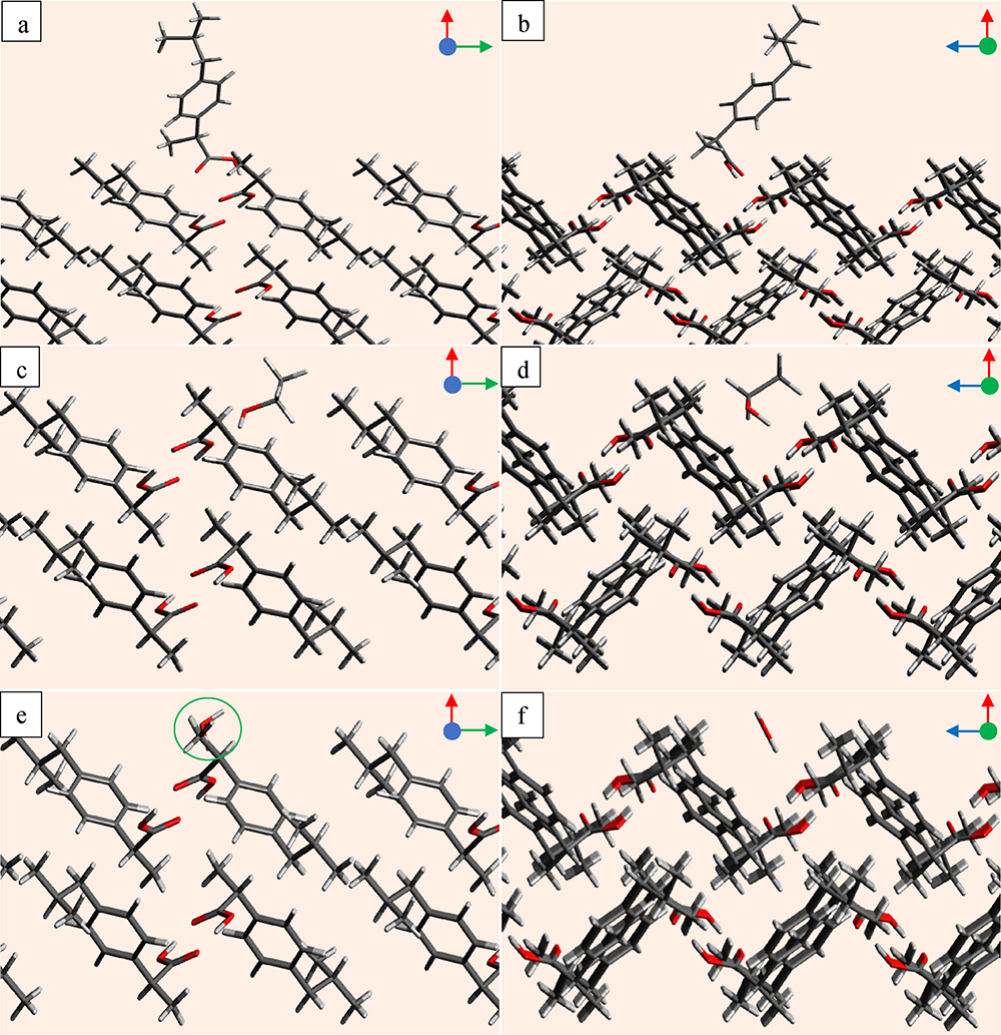
One of the many favorite probe positions on the face (002), where column 1 (a–e) shows the *uv* plane when viewed from the (−*w*) to (+*w*) direction and column 2 (b–f) shows the *uw* plane when viewed from (−*v*) to (+*v*) for (a, b) ibuprofen probe; (c, d) ethanol probe; and (e, f) water probe.

The interactions of the probe molecules with the face (002) are also visualized in two viewing directions using the Avogadro program, as shown in Figure 5. The one obvious difference between the faces (011) and (002) is the surface rugosity. The face (011) has a clear wide valley for molecules to land and reorient, whereas the face (002) is relatively smoother and does not offer a similar wide valley. The second difference between the faces is the two easily accessible carboxyl functional groups on the face (011), whereas, on the face (002), there is only one group in a very restricted position. Therefore, the opportunity for the probe molecules to make strong bonds on the face (011) is greater as compared to the face (002).

The ibuprofen probe is oriented in a favorable direction in Figure 5a,b to access the carboxyl group on the face (002) to make a strong hydrogen bond. This is the only suitable position where it could make a hydrogen bond, although it can have dispersive interactions at several positions over the reticular area. Similarly, the ethanol and water probes also get closer to the carboxyl group to make strong hydrogen bonds as shown in Figure 5c–f. The water molecule is circled in green in Figure 5c, as it is overlapped by a surface molecule in the viewing direction.

#### 3.1.3 Nature of Interaction Energies between Ibuprofen Surfaces and Probe Molecules

**Figure 6:**
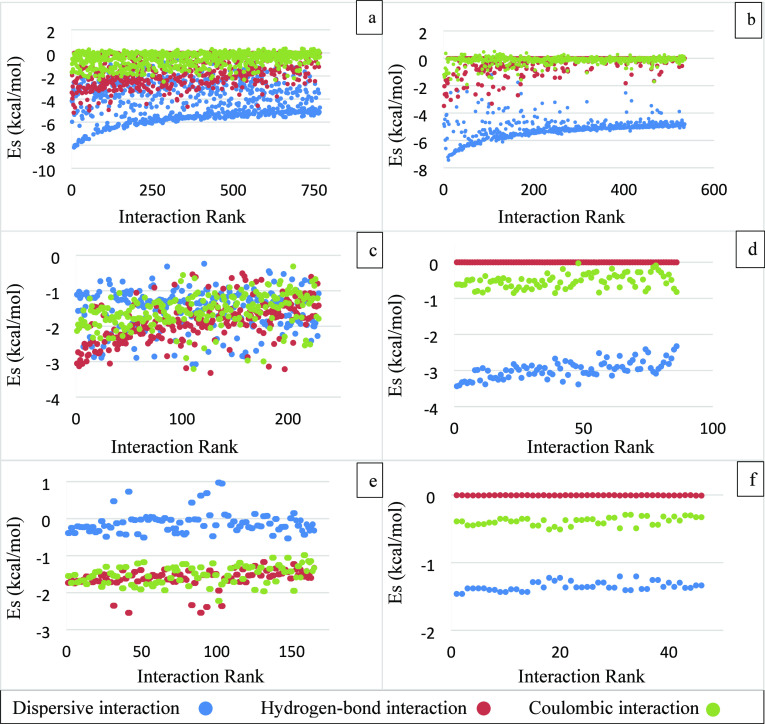
Contributions from dispersive, hydrogen bonding, and Coulombic interactions for (a, b) ibuprofen probe; (c, d) ethanol probe; and (e, f) water probe. The first column (a–e) is for the face (011) and the second column (b–f) is for the face (002).

The individual components of the surface interactions, including dispersive, hydrogen bonding, and Coulombic interactions, are plotted in Figure 6 for ibuprofen faces (011) and (002) and probes including ibuprofen, ethanol, and water. The contributions have a different scatter pattern for each probe and face. The strongest interactions between the ibuprofen probe and the face (011) are dispersive, with a significant contribution from hydrogen bonding interactions. The scatter is mixed with no clear boundaries as the ibuprofen probe can stack itself in a lateral direction on the face to maximize the strength of the dispersive interactions due to a wide valley and also have access to two carboxyl groups for hydrogen bonding interactions, which results in a mixed scatter. The interactions between the ibuprofen probe and the face (002) have a relatively smaller hydrogen bonding contribution, and the dispersive interactions contribute 92% in the interactions as shown in Table 1. Although the ibuprofen probe can orient itself to make hydrogen bonding interactions with the carboxyl group on the face (002) as shown in Figure 5a,b, due to restricted access, the strength of the interactions is relatively weaker.

**Table 1:** Percentage Energy Component Contributions in the Interactions Between Ibuprofen Faces (011) and (002) and the Ibuprofen, Ethanol, and Water Probes

	percentage contribution of the individual interaction energy components					
	F (011)			F (002)		
probe	dispersive	H-bond	Coulombic	dispersive	H-bond	Coulombic
ibuprofen	71.16	20.23	8.61	92.64	4.60	2.76
ethanol	29.61	38.33	32.06	85.16	0.01	14.82
water	3.30	49.75	46.95	77.44	0.29	22.27

The interactions of the ethanol probe with the face (011) produce a mixed scatter, where Coulombic interactions also contribute, along with the strong hydrogen bonding and dispersive interactions. The contribution of the dispersion interactions for the ethanol probe with the face (002) is greatest as shown by a separate scatter in Figure 6(d). The hydrogen bond interactions for this face are very small, making a straight line. It means that although the probe orientates itself to access the carboxyl group as shown in Figures 5(c) and (d), the hydrogen bonding interactions are still weak due to restricted access. The relative contribution of the hydrogen bonding and Coulombic interactions is nearly the same as shown in Table 1, for the face (011), whereas the contribution from the dispersive interactions is 85% for the face (002).

The water probe has weak interactions with faces (011) and (002), but the difference between the individual energy component contributions is clearer than other probes. The interactions on the face (011) have an approximately similar contribution from hydrogen bonding and Coulombic interactions, whereas the interactions with the face (002) are relatively weaker, where the major contribution of 77% is from dispersive interactions.

#### 3.1.4 Interaction Energy Landscapes of Ibuprofen Surfaces (011) and (002)

**Figure 7:**
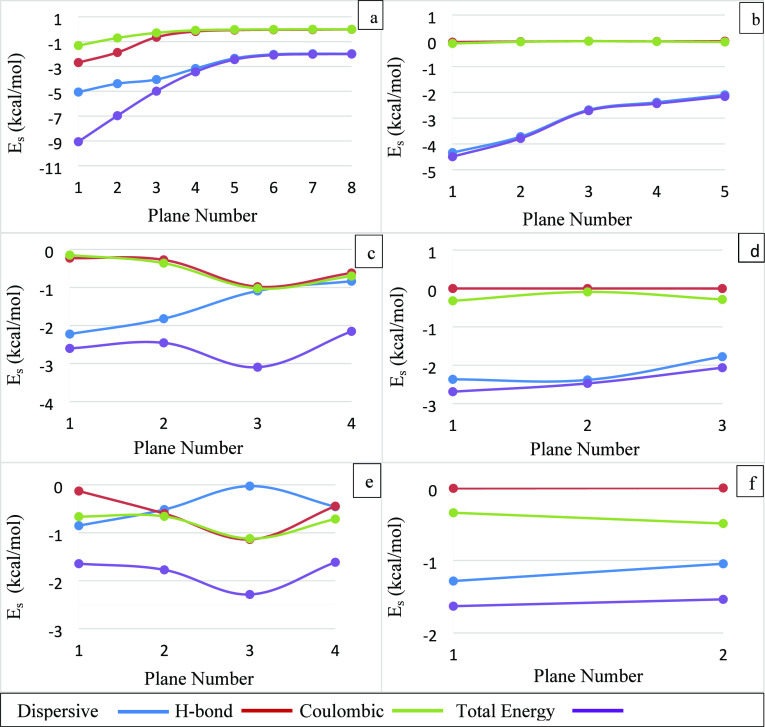
Average interactions over each plane plotted as a function of the plane number in the first column (a–e) for face (011) and in the second column (b–f) for the face (002) for (a, b) ibuprofen probe; (c, d) ethanol probe; and (e, f) water probe.

The interaction energies were averaged over each plane according to the plane schematic, as shown in Figure 1 and then plotted as a function of the plane number in Figure 7. The surface interactions between the ibuprofen probe and faces (011) and (002) differ in the first 5 Å distance, perpendicular to the faces, after that the interactions have similar energy distributions for both faces. First, hydrogen bonding interactions reach an asymptotic value and then the dispersive interactions for both faces. The dispersive interactions on the first plane in Figure 7a make the greatest contribution, and the strength of interactions weakens smoothly as the probe moves away from the surface toward plane 8. On the face (002), the total interactions have a relatively sharp transition from stronger to weaker interactions after plane number 3, due to restricted access to the carboxyl group on the reticular area. The Coulombic and hydrogen bonding interactions for the face (002) are very small compared to the face (011). Figure 7 reveals the energy landscape on the faces (011) and (002) in the parallel and perpendicular directions.

Figure 7c reveals that the interactions of the ethanol probe with the face (011) are bimodal as compared to the sigmoidal interactions of the ibuprofen probe with the same face. The interactions are stronger at plane 1, which is next to the carboxyl group inside the valley, as shown by the probe in Figure 4c. The strongest contribution is from the dispersive interactions at this point, as the ethanol probe interacts with both horizontally and vertically lying surface molecules. The opportunity for the dispersive interactions is a maximum at this plane because the probe is surrounded by the carbon chains of the host molecules. The probe then moves 1 Å away from plane 1 to plane 2 and the interactions weaken, but when it moves 1 Å further toward plane 3, it reaches the second carboxyl group, which has better access than the first carboxyl group to make strong hydrogen bonds. The deep well in total interaction energy on the face (011) is driven by strong hydrogen bonding interactions. Since no such valley is present on the face (002); therefore, the distribution of interaction energy of the ethanol probe with the face (002) is sigmoidal. The difference in the interactions of the probe with the faces (011) and (002) reveals a difference in the energy landscape.

The interactions of the water probe with the face (011) reveal a similar energy landscape to the ethanol probe; however, the dispersive interactions for the water probe are weaker than the ethanol probe. The interactions for the water probe with the face (002) are relatively weaker, and after plane 2, no interactions greater than the cut off value −1.5 kcal/mol are recorded.

The plane averaged plots between different faces and probe sizes suggest that to reveal the energy landscape, it is more suitable that only those interactions are captured by the probe, which are within a few angstroms of distance between the surface molecules and the probe. It is then possible to distinguish between the dispersive, hydrogen bonding, and Coulombic interactions more clearly to reveal the chemical and geometric features of the surface

The results in Figures 3–7 and Table 1 suggest that the surface interactions between the faces and probe molecules depend on the nature of the surface molecules, their orientation, and the surface rugosity. It can collectively be called an energy landscape. The strength of the surface interactions also depends on the nature of the solvent molecules, their size, and orientation. The surface interaction results in Figures 3–7 and Table 1 are similar to the results in the literature^19^ for ibuprofen surfaces and ibuprofen and ethanol probes.

#### 3.1.5 Validation of the Predicted Dissolution Rate Ratio (011)/(002)

The binding energies of the ibuprofen faces (011) and (002) as published in the literature^11^ are shown in the second column of Table 2. The averages of the surface interactions between the faces (011) and (002) and different probe molecules are given in the third column of Table 2. The surface interactions are then normalized by the molecular weights of the respective probe molecules, and normalized values of *E*_s_ are given in Table S1 of the Supporting Information. The energy differentials calculated from the normalized surface interactions are listed in Table 3. The energy differentials do not include the surface interactions between the ibuprofen faces and the water probe, as the interactions are weaker than the cutoff value of −2 kcal/mol. The binding energies in Table 2 are normalized by the molecular weight of the solute molecule and are given in Table S1. The modified binding energies calculated from eq 6 for faces (011) and (002) are given in Table 3 along with the energy differentials. The values of the surface rugosity, cell volume, and reticular area are given in Table S2.

**Table 2:** Binding Energies and Surface Interactions for the Ibuprofen Faces

ibuprofen faces	binding energy without surface interactions^1^ (kcal/mol Å)	surface interaction energies (kcal/mol)	probe
F (011)	–1.01	–5.86	ibuprofen
		–4.93	ethanol
		–3.25	water
F (002)	–0.78	–5.55	ibuprofen
		–3.49	ethanol
		–1.74	water

**Table 3:** Energy Differentials and Modified Binding Energies for Ibuprofen Faces

ibuprofen faces	*E*_d_ (kcal/mol)	*E*_b, mod_ (kcal/mol Å)
F (011)	–0.078	0.046
F (002)	–0.049	0.030

The predicted dissolution rate ratio (011)/(002) based on eq 7, by taking the ratio of the *E*_b, mod_ of the face (011) to the face (002), is 1.51, whereas the experimental ratio^11^ is 1.50 as given in Table 4. The discrepancy between the predicted and experimental dissolution rate ratio is 0.67%. Whereas, the discrepancy between the original binding energy model^11^ predicted and measured dissolution rate ratios (011)/(002) was 13.33%. The incorporation of the inequivalent wetting into the binding energy model by considering the interactions of the solution-state molecules with the ibuprofen faces (011) and (002) has improved the accuracy of prediction significantly.

**Table 4:** Validation of the Predicted Dissolution Rate Ratio (011)/(002)

dissolution rate ratio	based on *E*_b, mod_	experiment	percentage discrepancy
F (011)/F (002)	1.51	1.50	0.67

### 3.2 Application of the Modified Binding Energy Model to the Furosemide Water System

#### 3.2.1 Interaction Energies between Furosemide Surfaces and Probe Molecules

**Figure 8:**
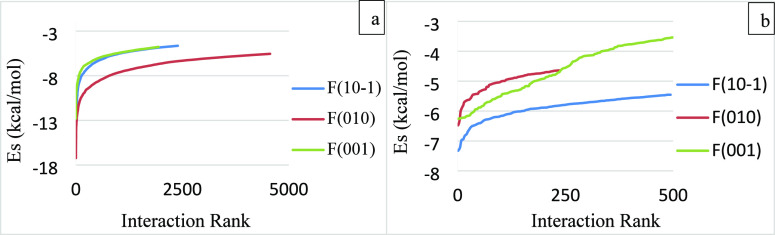
Interactions of the furosemide faces (101̅), (010), and (001) arranged in descending order with (a) furosemide probe and (b) water probe.

The surface interactions between the furosemide faces (101̅), (010), and (001) and the furosemide and water probes were carried out by the SystSearch method, and results for the interaction energies ranked on strength are presented as a function of the interaction rank in Figure 8. The full range of interactions is given in Figures S1 and S2 in the Supporting Information. The strongest interactions are observed between the face (010) and the furosemide probe, and the transition from stronger to weaker interactions is smooth. Large probes have been observed to produce a relatively smoother transition from stronger to weaker interactions. The furosemide probe has similar interaction curves with the faces (101̅) and (001), though the interaction rank is slightly greater, and interactions are slightly stronger for the face (101̅) at the transition point than the face (001). Unlike the furosemide probe, the water probe has the strongest interactions with the face (101̅), whereas it has the weakest interactions with the face (001).

#### 3.2.2 Favorable Orientations of Probe Molecules on Furosemide Crystal Surfaces

**Figure 9:**
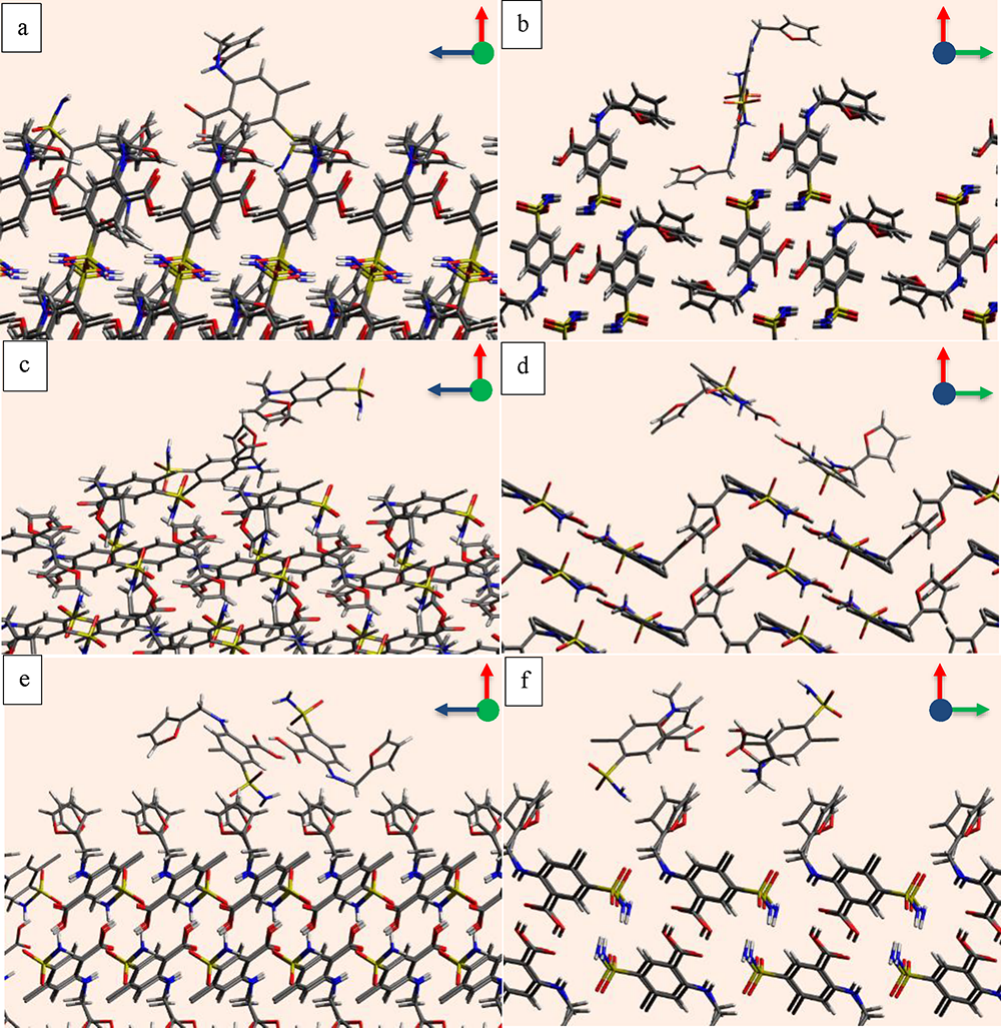
One of the many favorite probe positions for the furosemide probe on the (a) plane *uw* on the face (101̅) when viewed from (−*v*) to (+*v*); (b) plane *uv* on the face (101̅) when viewed from (−*w*) to (+*w*); (c) plane *uw* on the face (010) from (−*v*) to (+*v*); (d) plane *uv* on the face (010) from (−*w*) to (+*w*); (e) plane *uv* on the face (001) from (−*w*) to (+*w*); (f) plane *uw* from (−*v*) to (+*v*).

One of the many favorable orientations of the furosemide probe on the faces (101̅), (010), and (001) are shown in Figure 9. Since the furosemide asymmetric unit is made of two molecules as a dimer,^28^ so the search of surface interactions was carried out with a dimer probe. The face (101̅) has a narrow valley where the furosemide probe molecule has very restricted access as revealed in Figure 9a,b. The face (101̅) is rough, but not as the face (010), which has a wide-open valley where the furosemide probe can land and reorient to have strong interactions as shown in Figures 9c,d, and 8a. The interactions between the furosemide probe and the face (010) are therefore stronger than with the face (101̅). The face (001) is relatively smoother than the faces (101̅) and (010), with a narrow valley like the face (101̅). Therefore, the furosemide has a similar strength of interactions with the faces (101̅) and (001), but the interactions rank with the face (001) is smaller than the face (101̅).

**Figure 10:**
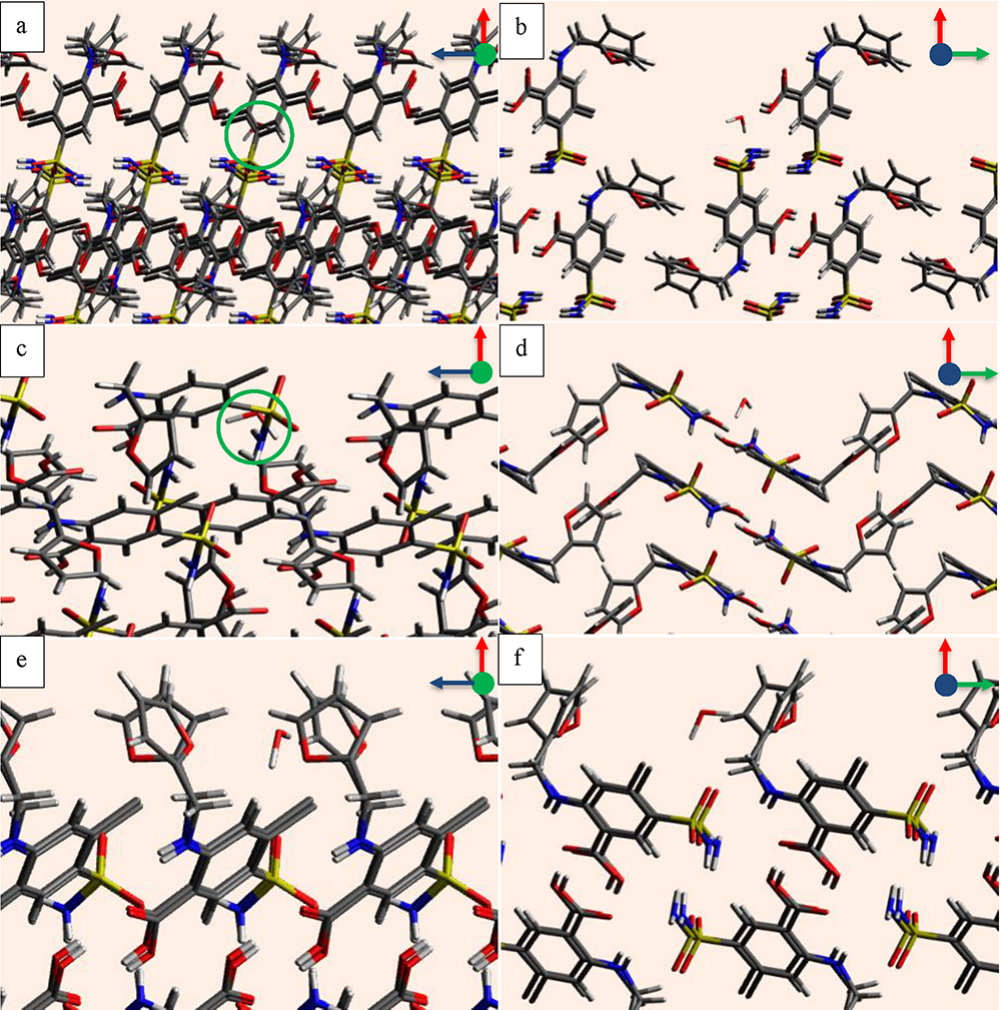
One of the many favorite probe positions for the water probe on the (a) plane *uw* on the face (101̅) when viewed from (−*v*) to (+*v*); (b) plane *uv* on the face (101̅) when viewed from (−*w*) to (+*w*); (c) plane *uw* on the face (010) from (−*v*) to (+*v*); (d) plane *uv* on the face (010) from (−*w*) to (+*w*); (e) plane *uv* on the face (001) from (−*w*) to (+*w*); (f) plane *uw* from (−*v*) to (+*v*).

The water probe has a smaller size; therefore, it lands inside the narrow valley on the face (101̅) to have strong interactions with the amino and sulfamoyl chloride functional groups as shown in Figure 10a,b. Since the number of functional groups exposed on the face (101̅) is greater than the faces (010) and (001), and the water molecule can access those functional groups in the valley, the water probe has strongest interactions with the face (101̅) as shown in Figure 8b.

The strength of the interactions of the water probe with the faces (101̅), (010), and (001) is according to the number of the functional groups on the faces. The probe interacts more strongly with the face (010) compared to the face (001) due to the greater number of exposed functional groups on the face (010) compared to the face (001). Though there are few stronger interactions with the face (001), they quickly converge to the cutoff value −2 kcal/mol.

#### 3.2.3 Nature of Interaction Energies between Probe Molecules and Furosemide Surfaces

The interactions of the furosemide surfaces with both probe molecules were split into the individual energy component contributions, and their scatters were plotted as a function of the interaction rank as shown in Figure S3 in the Supporting Information. The percentage contribution of each energy component is shown in Table 5, which suggests that the interactions between the furosemide probe and the faces (101̅), (010), and (001) of the furosemide crystal are dominated by the dispersive interactions.

**Table 5:** Percentage Contribution of the Individual Energy Components for the Interactions between the Furosemide Faces and Probe Molecules

	percentage contribution of the individual interaction energy components			
face	dispersive	H-bond	Coulombic	probe
F (101̅)	92.21	5.19	2.60	furosemide
F (010)	89.19	9.32	1.49	
F (001)	93.11	0.36	6.53	
F (101̅)	1.47	71.51	27.02	water
F (010)	–0.82	76.37	24.45	
F (001)	12.99	40.22	46.79	

There is a small hydrogen bonding contribution to the interactions of the probe with the faces (101̅) and (010), whereas there is a small contribution from the Coulombic interaction with the face (001). Like the ibuprofen probe in the ibuprofen case, most of the interactions of the furosemide probe with the furosemide faces are dispersive due to the large size of the probe and the presence of a carbon chain. However, the largest contribution to the interactions between the water probe and the furosemide faces is not dispersive but rather hydrogen bonding for the faces (101̅) and (010) and Coulombic interactions for the face (001).

There are five functional groups per reticular area that are accessible for a water probe on the faces (101̅) and (010) to make hydrogen bonds; therefore, the percentage contribution of the hydrogen bonding for both faces with water is greater. On the other hand, no functional group is directly exposed on the face (001); therefore, the contribution from hydrogen bonding and Coulombic interactions is nearly equal.

#### 3.2.4 Interaction Energy Landscapes of Furosemide Faces (101̅), (010), and (001)

**Figure 11:**
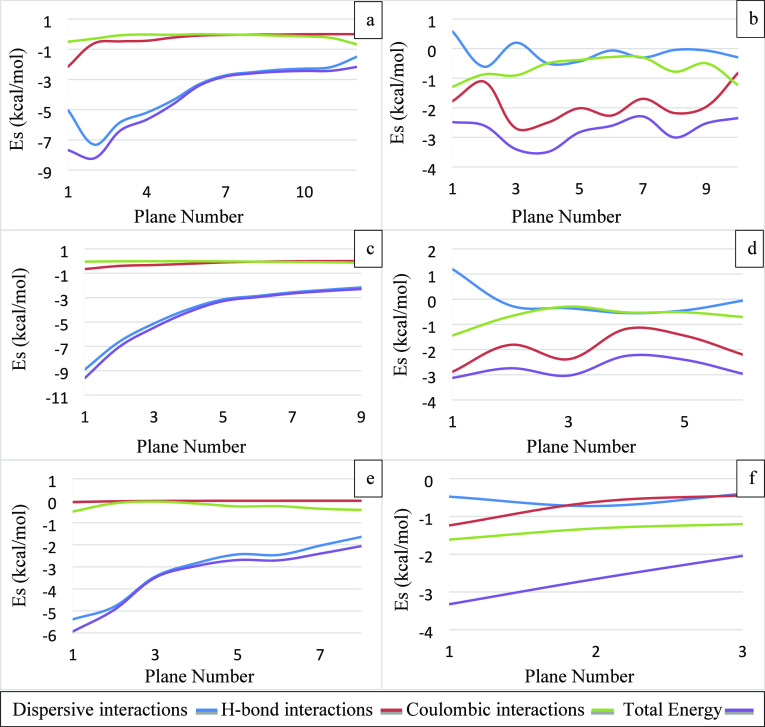
Furosemide surface interactions averaged over each plane and plotted as a function of the plane number between; (a) furosemide probe and face (10–1); (b) water probe and face (10–1); (c) furosemide probe and face (010); (d) water probe and face (010); (e) furosemide probe and face (001); and (f) water probe and face (001).

The surface interactions between the furosemide faces (101̅), (010), and (001) and the probe molecules including furosemide and water were averaged over each grid plane and plotted as a function of the grid plane number as shown in Figure 11. The major contributing energy component for the interactions between the furosemide faces and the furosemide probe is dispersive in each plane. This is expected due to the large size of the furosemide molecule. The interactions between the furosemide on each plane with all faces weaken as the probe moves away from the faces, and no reversal in the curve trends is observed.

For the interactions between the water probe and the furosemide faces (101̅) and (010), the hydrogen bonding has a maximum contribution on each plane. This is according to the functional groups on the faces, as shown in Figures 9 and 10. Several functional groups are present at different distances from the first grid plane on the reticular area of each face. The curves in Figure 11d are relatively smoother due to fewer functional groups on the face as compared to the face (101̅). The surface interactions of the water probe show straight lines on each grid plane for face (001). This is expected, as there is no single dominant interaction contribution for this face with the water probe. The water probe interactions with the furosemide faces (101̅), (010), and (001) reveal the energy landscape in a relatively clearer way than the furosemide probe due to the smaller size of the water probe.

#### 3.2.5 Validation of the Predicted Dissolution Rate Ratios (101̅)/(001) and (010)/(001)

The solid-state binding energy values for the furosemide faces (101̅), (010), and (001) obtained in our previous study^11^ are given in Table 6 along with the average surface interaction values for the same faces calculated from the SystSearch method in this study. The surface interactions normalized by the molecular weight of the probe molecules and the binding energies normalized by the molecular weight of the furosemide are given in Table S3. The energy differentials calculated from eq 5 are given in the second column of Table 7, whereas the modified binding energy values calculated from eq 6 are given in the third column of Table 5. The values of the reticular area, cell volume, and the surface rugosity used in eq 6 are given in Table S4. The experimental face-specific dissolution rates for the faces (101̅), (010), and (001) of the furosemide taken from the literature^2^ are given in the fourth column of Table 7.

**Table 6:** Values of the Binding Energies and Surface Interactions for Furosemide

face	binding energy, *E*_b_ (kcal/mol Å)	surface interactions, *E*_s_ (kcal/mol)	probe
F (101̅)	–0.38	–5.77	furosemide
F (010)	–0.03	–6.91	
F (001)	–0.01	–5.76	
F (101̅)	–0.38	–5.89	water
F (010)	–0.03	–5.06	
F (001)	–0.01	–4.08	

**Table 7:** Energy Differentials, Modified Binding Energies and Experimental Dissolution Rates of Furosemide Faces

faces	*E*_d_ (kcal/mol)	*E*_b,mod_ (kcal/mol Å)	experimental dissolution rates^2^ (μmol/s m^2^)
F (101̅)	–0.31	0.128	16.1 ± 6.7
F (010)	–0.26	0.147	12.6 ± 6.9
F (001)	–0.21	0.026	2.8 ± 1.4

The face-specific relative dissolution rate ratios (101̅)/(001) and (010)/(001) were calculated from the modified binding energy values and compared with those determined using the experimental data^2^ in Table 8. The predicted dissolution rate ratio (101̅)/(001) is 4.91, whereas the experimental value is 5.75. The discrepancy between the predicted and the experimental dissolution rate ratios is 14.6%, whereas the discrepancy between the measurement and prediction from the original binding energy model was approximately 499%.^11^ As in the case of ibuprofen, the incorporation of the inequivalent wetting of the crystal faces has reduced the discrepancy, but in this case, the reduction is very large from 499% to 14.6%, resulting in a significant improvement in the quality of prediction.

**Table 8:** Validation of the Predicted Dissolution Rate Ratios for Furosemide

dissolution rate ratios	based on *E*_b,mod_	experiment	percentage discrepancy
(101̅)/(001)	4.91	5.75	14.6
(010)/(001)	5.62	4.50	24.8

The dissolution rate ratio (010)/(001) predicted by the modified binding energy model is 5.62, whereas the experimental ratio is 4.50, with a discrepancy of 24.8%. This is acceptable considering a large standard deviation in the experimental results, which is approximately 50% for each data point.^2^ The discrepancy between the predicted and measured dissolution rate ratios (010)/(001) was 31.33% for the original binding energy model.^11^

The method developed in this study provides an effective approach to correlating crystal shape with its dissolution behavior, thereby enhancing the accuracy of dissolution rate predictions. This method can facilitate a rapid evaluation of how morphological changes affect dissolution. This study demonstrates the critical role of face-specific dissolution rates in determining the overall dissolution rate. The method’s computational efficiency makes it a valuable tool for optimizing the design of active pharmaceutical ingredient (API) crystal morphology to achieve target bioavailability in solid dosage forms. To ensure its wider applications, it is prudent to assess the robustness of the method for other dissolution systems from the literature.

## 4 Conclusions

An improved binding energy model to predict the face-specific relative dissolution rates has been developed by incorporating both the intermolecular interactions in the solid-state structure of the crystals and the interfacial intermolecular interactions between the crystal faces and the solute-state molecules. This model is a modification of a previous binding energy model,^11^ where surface interactions with the solution-state molecules were not considered under equivalent wetting assumption. To incorporate the interfacial synthons, the surface interactions between different crystal surfaces and probe molecules have been investigated in detail using the calculated results obtained from a grid based SystSearch method. This model is applied to predict the face-specific relative dissolution rates of the ibuprofen and furosemide single crystals in 95% v/v ethanol–water and an aqueous medium, respectively, and the predictions are compared with the experimental data.^2,11^

The images for the most favorable surface interaction orientations for the probe molecules on different faces of the furosemide and ibuprofen crystals revealed that the surfaces interact with solution state molecules differently due to the differences in the surface rugosity and orientation of the surface molecules. It also suggested that the relative size difference between the reticular areas of different faces affects the interaction rank. The individual energy component scatters revealed that the interactions for the larger probes were generally dominated by the dispersive interactions, whereas for the small probes, the percentage contribution from the dispersive interactions was significantly smaller. The plane averaged interactions as a function of the grid plane number suggest that the energy landscape differs due to the variation of surface rugosity and the exposure of the functional groups. The energy landscape information was revealed more clearly by smaller size probe. The results indicated that very small molecules or single atoms are more useful to calculate the geometric features of the surfaces, such as the sizes of the peaks and valleys.

The dissolution rate ratio (011)/(002) for ibuprofen calculated by the modified binding energy model improved the accuracy as compared with the original binding energy model. The discrepancy between the prediction and experiment was reduced from 13.33 to 0.67%. For furosemide, the discrepancy between the predicted and the experimental dissolution rate ratios (101̅)/(001) was reduced from 499 to 14.6% and that for the dissolution rate ratios (010)/(001) from 31.33 to 24.8%. This suggested that the solution-state molecules play an important role in the anisotropic face-specific dissolution rates of the single faceted-crystals. The geometric features, such as the cell volume, reticular area, and surface rugosity also play a role, but they have not been explored in further detail in this study.

The modified binding energy model is a powerful tool that is an alternative approach to computationally intensive MD simulations. It approximates the MD simulations in a computationally expedient way. The modified binding energy model successfully predicted the face-specific dissolution rate ratios for two different dissolution systems, ibuprofen and furosemide, with good accuracy. This modeling approach can be used reliably for the digital design of API crystal morphology in order to achieve desired bioavailability of solid doge forms. The robustness of this model can be further tested by applying it to different dissolution systems studied experimentally in case-studied compounds from the literature.

## Supporting Information Available

Normalized binding energy and surface interactions for ibuprofen, geometric values of the reticular area, cell volume and surface rugosity for ibuprofen, normalized binding energy and surface interactions for furosemide, geometric values of the reticular area, cell volume and surface rugosity for furosemide, surface interactions of the furosemide and water probes with the furosemide faces (101̅), (010), and (001), all of the captured surface interaction energies vs interaction ranks plots for furosemide faces and probe molecules, and scatters of the contribution of the individual energy components including dispersive, h-bond, and Coulombic to the surface interactions of furosemide with furosemide and water probes for the faces (101̅), (010), and (001) (PDF)
