# Supplementary material for: Impact of Inequivalent Wetting on the Face-Specific Dissolution Rates for Single Faceted-Crystals Predicted from Solid-State Binding Energies
Source: Cryst Growth Des. 2024 Jun 10;24(12):4894–905. doi: 10.1021/acs.cgd.2c00043 (PMC11191405; doi:10.1021/acs.cgd.2c00043)
Supplement: Supplementary file 1 — cg2c00043_si_001.pdf [file cg2c00043_si_001.pdf]

Supporting Information

Impact of Inequivalent Wetting on the Face-Specific Dissolution Rates for Single Faceted-Crystals Predicted from Solid-State Binding Energies

Muhammad Najib [a], Robert B. Hammond [a], Tariq Mahmud [a], Toshiko Izumi [b]*

[a]School of Chemical and Process Engineering, The University of Leeds, Leeds LS2 9JT, UK

[b]Pfizer Research & Development UK, Ramsgate Road, Sandwich, Kent, CT13 9NJ, UK

Table S1. Normalised binding energies and surface interactions for ibuprofen

Face	Normalised Binding Energy, E_b (kcal/mol.Å)	Normalised Surface Interactions, E_s (kcal/mol)	Probe
F (011)	-0.0049	-0.107	Ibuprofen
		-0.076	Ethanol
		-0.029	Water
F (002)	-0.0038	-0.027	Ibuprofen
		-0.180	Ethanol
		-0.097	Water

Table S2. Geometric values for the ibuprofen including reticular area, cell volume and surface rugosity

Face	Reticular area (Å^2)	Cell volume (Å^3)	Surface rugosity
F (011)	193.62	1224.54	4.08
F (002)	231.33	1224.54	3.71

Table S3. Normalised binding energies and surface interactions for furosemide

Face	Normalised binding Energy, E_b (kcal/mol)	Normalised surface Interactions, Es (kcal/mol)	Probe
F (10$\bar{1}$)	-1.1E-03	-0.017	Furosemide
F (010)	-1.0E-04	-0.021	
F (001)	-3.4E-05	-0.017	
F (10$\bar{1}$)	-1.1E-03	-0.33	Water
F (010)	-1.0E-04	-0.28	
F (001)	-3.4E-05	-0.23	

Table S4. Geometric values for the furosemide including reticular area, cell volume and surface rugosity

Face	Cell volume (Å^3)	Reticular area (Å^2)	Surface rugosity
F (10$\bar{1}$)	1324.84	157.15	3.52
F (010)	1324.84	143.95	5.19
F (001)	1324.84	90.40	1.83

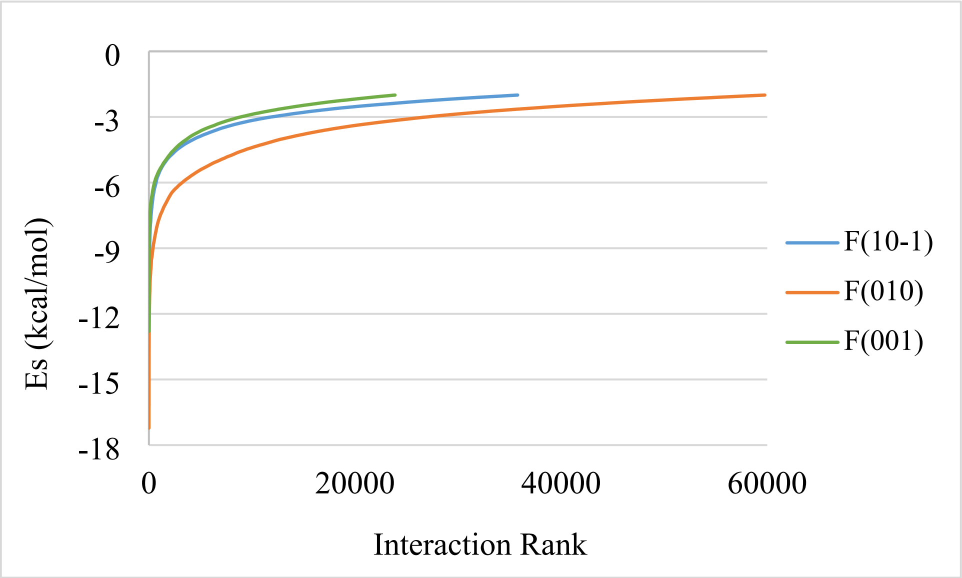

Figure S1. The interactions between the furosemide faces (10$\bar{1}$), (010) and (001) and the furosemide probe arranged in descending order as a function of the interaction rank.

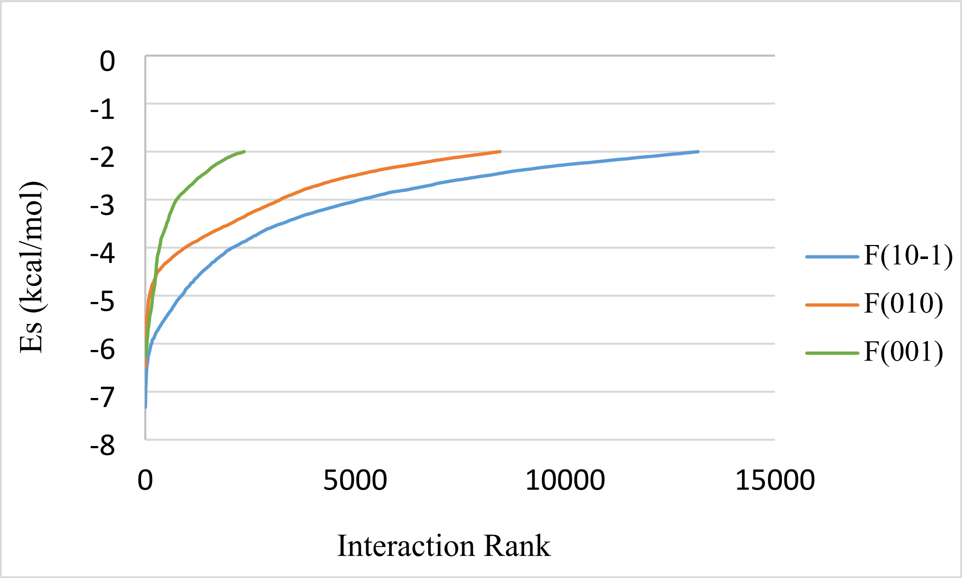

Figure S2. The interactions between the furosemide faces (10$\bar{1}$), (010) and (001) and the water probe arranged in descending order as a function of the interaction rank.

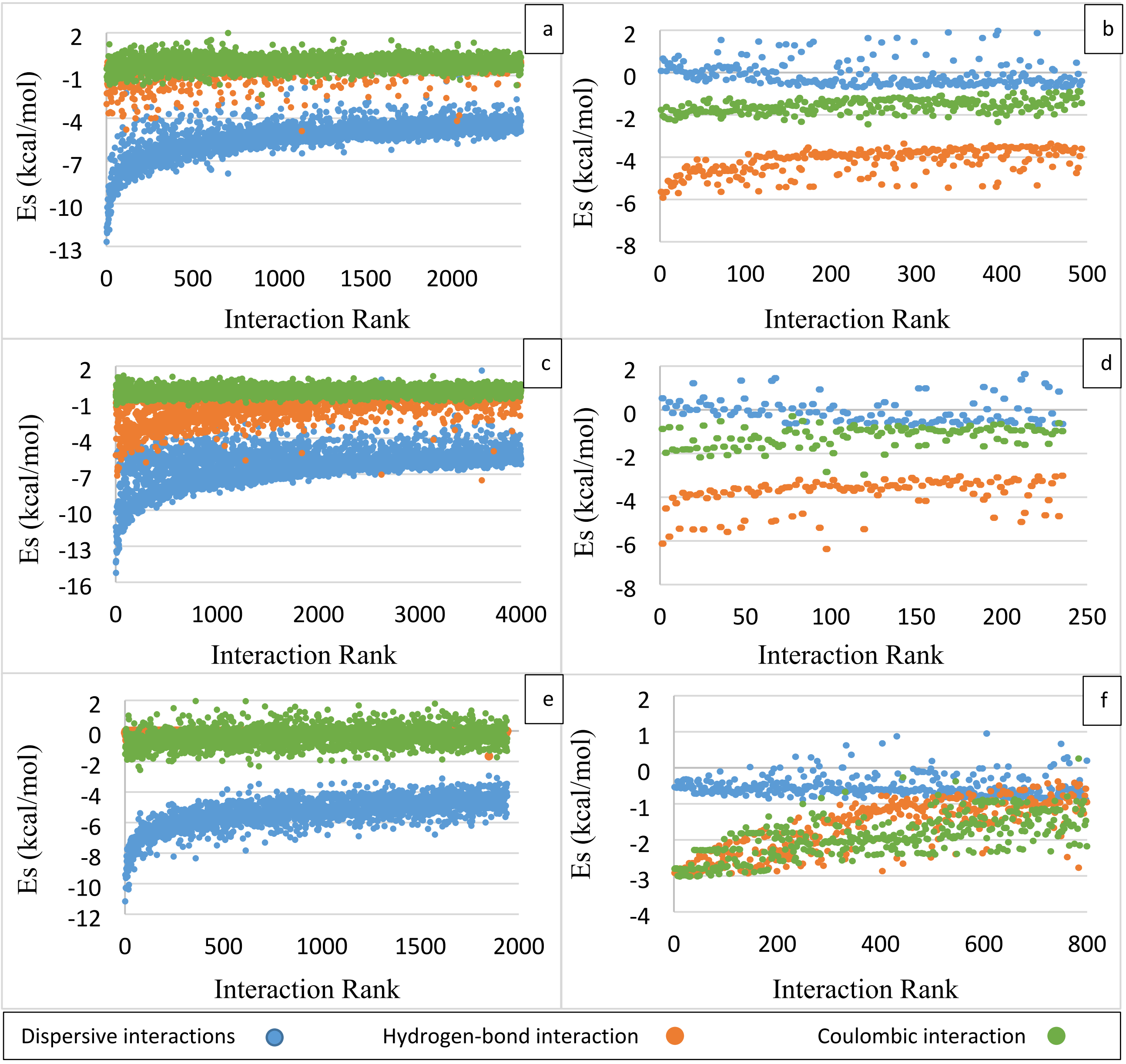

Figure S3. Individual energy components as a function of the interactions rank in the surface interactions between; (a) furosemide probe and face (10$\bar{1}$); (b) water probe and face (10$\bar{1}$); (c) furosemide probe and face (010); (d) water probe and face (010); (010); (e) furosemide probe and face (001); (f) water probe and face (001).

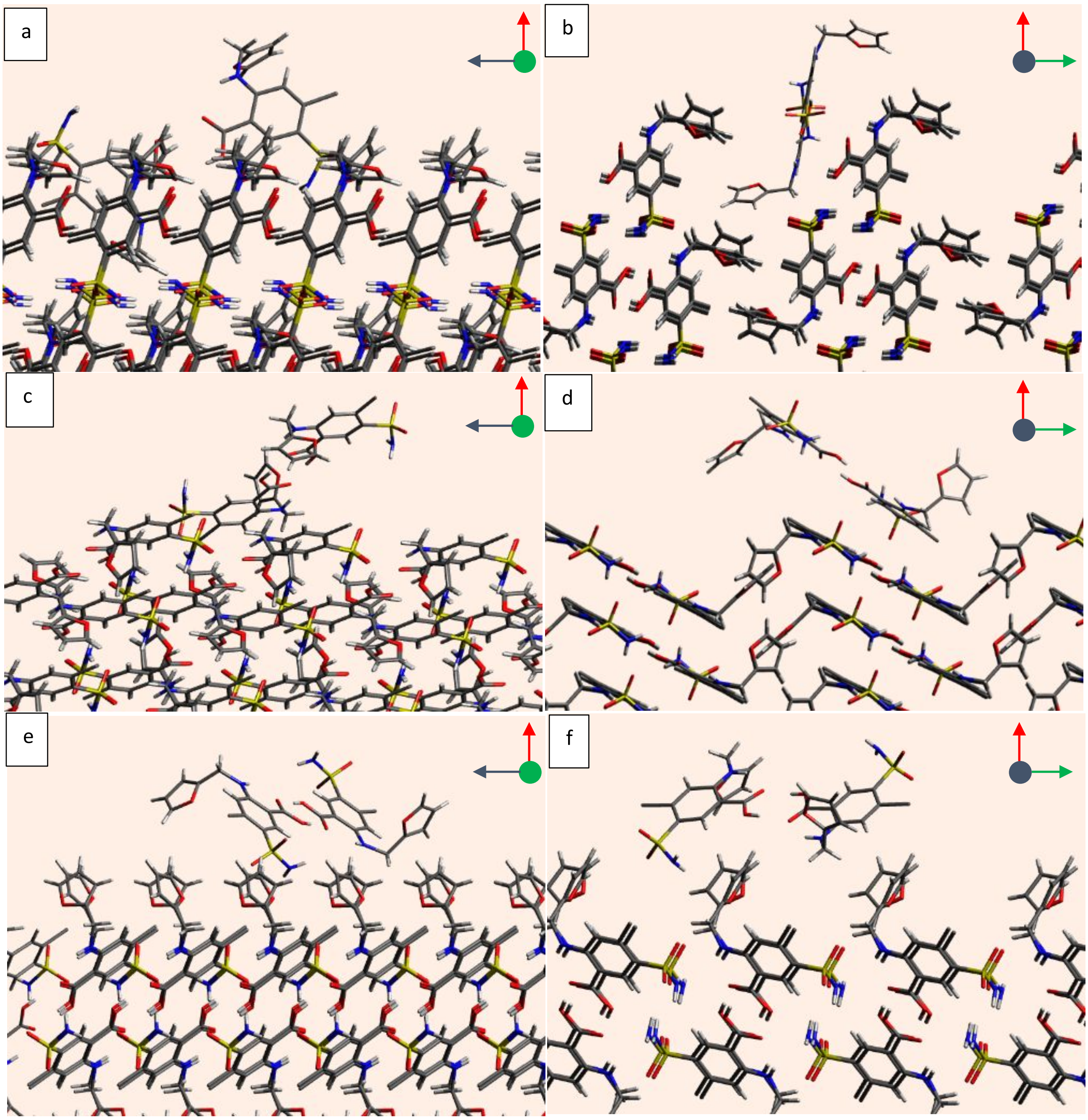

Figure S4. One of the many favorite probe positions for the furosemide probe on the; (a) plane uw on the face (10$\bar{1}$) when viewed from (-v) to (+v); (b) plane uv on the face (10$\bar{1}$) when viewed from (-w) to (+w); (c) plane uw on the face (010) from (-v) to (+v); (d) plane uv on the face (010) from (-w) to (+w); (e) plane uv on the face (001) from (-w) to (+w); (f) plane uw from (-v) to (+v).

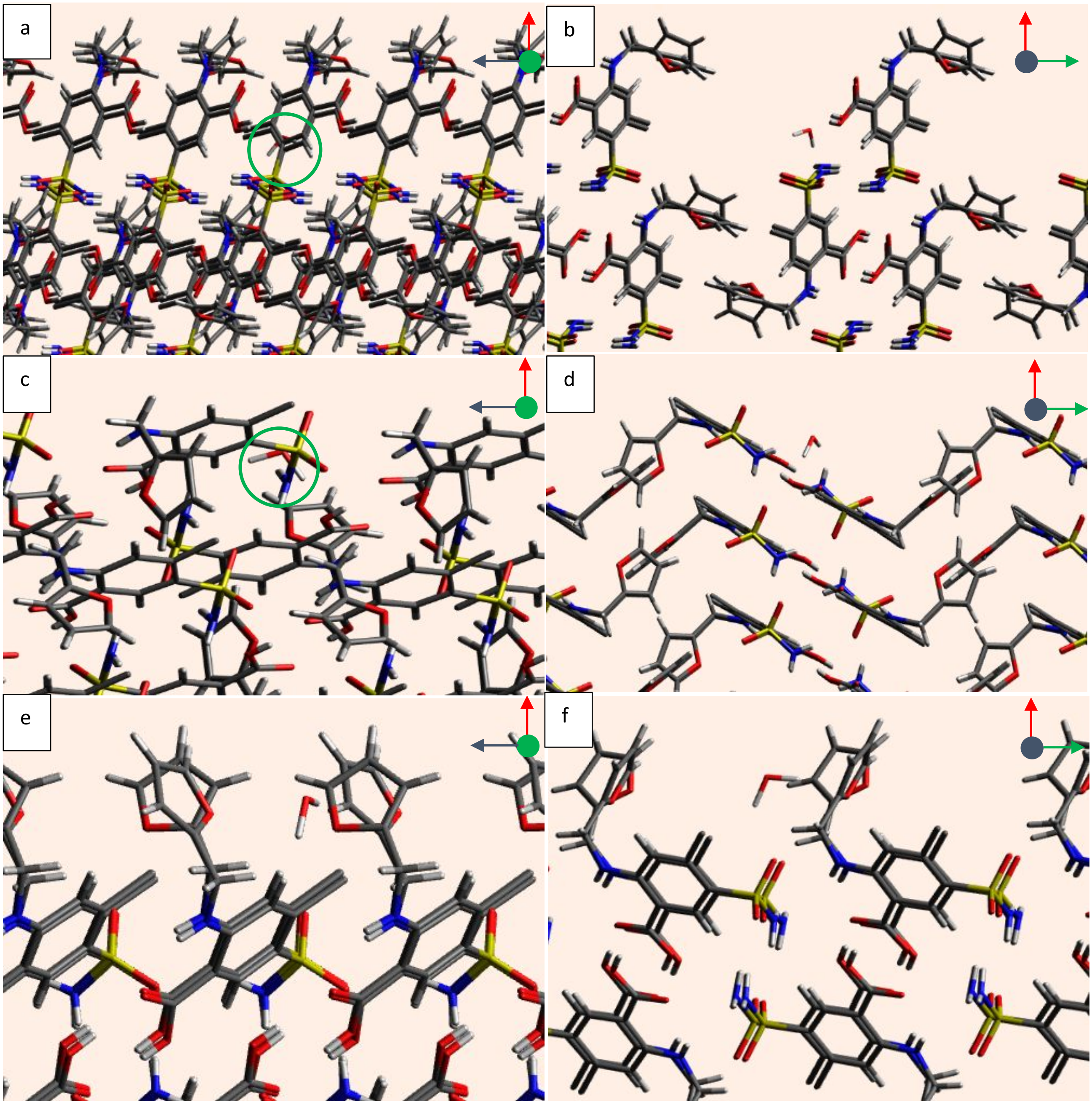

Figure S5. One of the many favorite probe positions for the water probe on the; (a) plane uw on the face ($10\bar{1}$) when viewed from (-v) to (+v); (b) plane uv on the face ($10\bar{1}$) when viewed from (-w) to (+w); (c) plane uw on the face (010) from (-v) to (+v); (d) plane uv on the face (010) from (-w) to (+w); (e) plane uv on the face (001) from (-w) to (+w); (f) plane uw from (-v) to (+v).
